# Advancing Glaucoma Treatment During Pregnancy and Breastfeeding: Contemporary Management Strategies and Prospective Therapeutic Developments

**DOI:** 10.3390/biomedicines12122685

**Published:** 2024-11-25

**Authors:** Maria Letizia Salvetat, Mario Damiano Toro, Francesco Pellegrini, Paolo Scollo, Roberta Malaguarnera, Mutali Musa, Liliana Mereu, Daniele Tognetto, Caterina Gagliano, Marco Zeppieri

**Affiliations:** 1 Department of Ophthalmology, Azienda Sanitaria Friuli Occidentale, Via Montereale 24, 33170 Pordenone, Italy; mlsalvetat@hotmail.it; 2 Eye Clinic, Public Health Department, Federico II University, Via Pansini 5, 80131 Naples, Italy; 3 Department of Special Surgery, University of Jordan, Queen Rania St, Amman 11942, Jordan; 4 Department of Medicine and Surgery, University of Enna “Kore”, Piazza dell’Università, 94100 Enna, Italy; 5 Gynecology and Obstetrics Unit, Cannizzaro Hospital, 95126 Catania, Italy; 6 Department of Optometry, University of Benin, Benin City 300238, Edo State, Nigeria; 7 Gynecology and Obstetrics Unit, Policlinico G. Rodolico, Department of General Surgery and Medical-Surgical Specialties, University of Catania, 95124 Catania, Italy; 8 Department of Medicine, Surgery and Health Sciences, University of Trieste, 34127 Trieste, Italy; 9 Mediterranean Foundation “G.B. Morgagni”, 95125 Catania, Italy; 10 Department of Ophthalmology, University Hospital of Udine, 33100 Udine, Italy

**Keywords:** intraocular pressure, ocular hypertension, glaucoma, anti-glaucoma medications, glaucoma in pregnancy, glaucoma in breastfeeding, glaucoma laser and surgery in pregnancy, fetal development, teratogenic effect, congenital disabilities

## Abstract

The management of glaucoma in pregnancy and breastfeeding requires a careful evaluation of treatment choices to guarantee the well-being of both the mother and the developing fetus. This review explores the intricacies of controlling glaucoma in pregnant and breastfeeding women, including a comprehensive overview of existing glaucoma treatment methods, clinical guidelines, and future therapeutic approaches. The efficacy and safety profiles of traditional treatment approaches, such as topical and systemic medicines and surgical treatments, are evaluated specifically about their use during pregnancy and breastfeeding. The significance of personalized treatment programs to achieve a balance between controlling intraocular pressure and ensuring the safety of the fetus and the newborn and the importance of a multidisciplinary approach that includes ophthalmologists, obstetricians, and other healthcare experts are underlined. Non-pharmacological therapies, lifestyle adjustments, and the importance of patient education in the management of glaucoma during pregnancy and the post-partum period are also examined. Advancing our comprehension of and strategy toward glaucoma can reduce the effects of glaucoma on maternal, fetal, and newborn well-being.

## 1. Introduction

Glaucoma is defined as a multifactorial, chronic, degenerative, and progressive optic neuropathy (ON) [1]. It represents the leading cause of permanent blindness worldwide, and its prevalence has been estimated to drastically enhance in the future because of the increasing aging of the population [1]. Despite identifying multiple genetic and environmental risk factors for its onset and progression, ocular hypertension (OHT) is considered the most important and the only modifiable one [2]. A reduction in intraocular pressure (IOP) represents the most important target of various available therapeutic options [1]. Given that it usually affects the elderly population [1], glaucoma in childbearing age is relatively uncommon [3]. It is expected to become more frequent in this subgroup of patients because of the increasing maternal age over recent decades [3].

The management of glaucoma in planning-to-be-pregnant, pregnant, and breastfeeding women presents specific challenges for ophthalmologists for several reasons [4,5,6]. First of all, it requires a delicate balance between the risk of glaucomatous damage progression in the mother and the possible side effects of the different therapeutic options for the fetus and newborn child [7,8]. Secondarily, glaucoma detected during childbearing age is likely to be classified as congenital, juvenile, associated with anterior segment dysgenesis, or secondary to uveitis, trauma, diabetes, surgery, pseudoexfoliation capsule, or pigment dispersion syndrome [3]. Unfortunately, all these types of primary or secondary glaucomas are known to be refractory to conventional treatments [1,9]. Moreover, considering that ethical and legal constraints prevent performing large, prospective, randomized, controlled trials (RCTs) evaluating drug efficacy and safety in pregnant and breastfeeding patients, the potential teratogenicity of most human drugs remains largely unknown [10]. In particular, no topical or systemic IOP-lowering medications have evidence of safety for the mother and fetus based on human studies [11,12]. The absence of RCT-based precise guidelines helping physicians in making therapeutic decisions induces a general level of uncertainty regarding the treatment of glaucomatous pregnant or nursing patients inside the ophthalmological community [13].

The present review distinguishes itself from the current literature because it offers a wide overview of the more recent knowledge about the influence of pregnancy, breastfeeding, labor, and delivery on the visual system and the glaucomatous disease course. The benefits and side effects of all currently available and futuristic medical, laser, and surgical treatments to manage ocular hypertension and glaucoma in pregnant and nursing women will be presented, discussed, and summarized in an easy-to-consult recommendations section. A particular emphasis will highlight the need for an extremely individualized, consensual, and multidisciplinary therapeutic approach in order to enhance both maternal and fetal health outcomes.

## 2. Visual System Changes in Healthy Women During Pregnancy, Labor, Delivery, and Breastfeeding

Pregnancy is a complex physiological process characterized by typical circulatory system modifications, the rearrangement of the abdominal organs’ positions, and a massive fluctuation in hormonal levels involving placental and maternal endocrine glands and fetal adrenal gland hormones. These changes may potentially lead to changes in all the body’s organs, including the eye and the entire visual system [14,15,16]. The visual changes occurring during pregnancy can be divided into physiological, which reverse after delivery or with the cessation of breastfeeding, and pathological, which may require prompt treatment or may induce permanent damages [14,15,16]. Pregnancy prompts various physiological modifications, such as typical brown pigmentation of the eyelids (pregnancy chloasma); dry eye syndrome; reduced corneal sensitivity; corneal subedema with increased central corneal thickness (≈3%); decreased corneal rigidity; increased anterior chamber and sclero-corneal angle depth; increased corneal and lens curvature radius; reduced mean intraocular pressure (IOP) and IOP fluctuations; enhanced ocular blood flow; reduced mean retinal sensitivity on visual field tests; possible visual field defects from pituitary gland chiasmatic compression [14,15,16].

Pathological alterations may encompass pre-existing ocular pathologies modified by pregnancy, such as keratoconus, glaucoma, diabetic retinopathy, neuro-ophthalmic pathologies, etc.; diseases occurring for the first-time during pregnancy, including keratoconus, dry eye syndrome, central serous chorioretinopathy, etc.; or ocular manifestations of systemic diseases that are specific to (increase of the pituitary gland volume, pre-eclampsia and eclampsia) or more frequent during pregnancy (idiopathic intracranial hypertension, disseminated intravascular coagulation, cerebral venous thrombosis, etc.), such as papilledema, hypertensive retinopathy, retinal vascular occlusions, exudative retinal detachment, and compressive or ischemic visual field defects [14,15,16].

Pregnant women may be particularly susceptible to drug- or infection-related complications, as examined in by Chang et al. [17] or Li et al. [18], which highlights the imperative for comprehensive and flexible management strategies in these patients.

Amongst physiological modifications, it has been demonstrated that healthy pregnant women typically show lower mean IOP values and IOP fluctuations than age-matched non-pregnant ones [19,20], with an IOP decrease significantly higher in multigravida [21]. The IOP reduction begins at approximately 18 weeks of gestation; it increases progressively during pregnancy, reaching a maximum reduction of 1–4 mmHg during the third trimester and early post-partum period, when the IOP difference between pregnant and non-pregnant women becomes statistically significant; and returns to baseline values approximately 2–3 months after delivery. Additionally, a significantly reduced diurnal IOP fluctuation has been found during pregnancy, with a range approximately 50% lower in pregnant than in non-pregnant women [19,20].

The mechanisms explaining lowering IOP during pregnancy remain controversial (Table 1). They are supposed to be due to pregnancy-induced electrolytic modifications and high levels of estrogens, progesterone, relaxin, and beta human chorionic gonadotropin, whose concentrations have indeed demonstrated to be inversely related to IOP measurements; moreover, they seem mainly associated with an increased aqueous outflow, instead of a downregulation of aqueous production [22,23]. Furthermore, the corneal subedema, due to the water retention and the reduced corneal rigidity found in pregnant women [14,15,16,19,20], may induce an underestimation of the IOP values taken with several tonometric methods, especially those based on corneal applanation [24]. This issue is still debated: other studies did not find any statistically significant modification of corneal thickness or biomechanics during pregnancy [25].

Considering labor- and delivery-induced IOP changes, previous authors have shown that the mean IOP increases approximately 1–2 mmHg during vaginal labor, decreases by 3 mmHg immediately after delivery, and returns to pre-labor levels 3 days after delivery [26]. On the other hand, the fundal pressure used to facilitate delivery during Cesarian (C)-section is associated with a small IOP increase of 3–4 mmHg [27]. IOP modifications during both vaginal delivery and C-section are thought to be not clinically significant [26,27].

## 3. Glaucoma During Pregnancy and Breastfeeding

### 3.1. Prevalence and Types of Glaucoma in Pregnant and Breastfeeding Women

Although mostly associated with an older population, OHT and glaucoma may also affect women during their childbearing age, pregnancy, and breastfeeding, with a prevalence that is thought to likely increase in the future for several reasons: the enhanced maternal age related to advances in reproductive technologies, the greater consciousness of the disease, and new methods that allow for an early glaucoma diagnosis [3].

The specific incidence and prevalence of glaucoma during pregnancy and lactation are unknown. However, epidemiological studies have estimated that, in women of childbearing age, the prevalence of glaucoma of all types and primary open-angle glaucoma (POAG), i.e., the most frequent glaucoma subgroup, may be approximately 1% and 0.5%, respectively [28,29,30].

Glaucoma in pregnancy and breastfeeding seems to be less rare than expected. In a large survey conducted in the United Kingdom in 2007 involving 282 ophthalmologists, more than 1/4 of the participants declared that they had experience treating glaucoma in pregnant or nursing patients [13]. Considering that primary open-angle or angle-closure glaucomas typically affect patients older than 60 years [1], glaucoma detected in women during their childbearing age is likely to be classified as one of the following subgroups: congenital glaucoma, glaucoma associated with anterior segment dysgenesis (Axenfeld–Rieger syndrome, Sturge–Weber syndrome, Peter’s anomaly, aniridia, iridocorneal endothelial (ICE) syndrome, etc.); juvenile open-angle glaucoma (JOAG); glaucoma secondary to uveitis, trauma, diabetes, or post-surgery (post-penetrating keratoplasty or retinal surgery); glaucoma in capsular pseudoexfoliation or pigment dispersion syndrome [3]. Unfortunately, all these glaucoma types are known to be refractory to conventional treatments [9].

### 3.2. IOP Changes and Glaucoma Clinical Course During Pregnancy and Breastfeeding

Several studies have demonstrated that the characteristic IOP decrease found in healthy women during pregnancy [19,20] is likely to occur even more in pregnant patients with pre-existing OHT or glaucoma. Its amount seems directly proportional to the gestational age [31,32]. In addition to their IOP-lowering effect [22], the high levels of estrogens typically found in pregnancy seem to have an IOP-independent protective role against glaucomatous damage development and progression. Large population studies have found that a greater estrogen life exposure because of early menarche or late menopause [33] and the assumption of post-menopausal hormone replacement therapy (estrogens alone or in combination with progesterone) [34] are significantly associated with a lower risk of POAG. The protective role of estrogens against glaucoma is still under investigation and may be related to different mechanisms, including their vasodilatory effect, with increasing ocular blood flow; the activation of collagen synthesis, which may reduce the deformability of the lamina cribrosa and the optic nerve axon compression at its level; and their neuroprotective effect on the retinal ganglion cells, as demonstrated in animal models (Table 1) [35,36].

Despite all these general favorable conditions, IOP and glaucomatous damage progression during pregnancy remain unpredictable. Although IOP values appear stable or lower in many cases [31,32], and thus the need for IOP-lowering medication is reduced, 70–85% of OHT or glaucomatous patients still require medical therapy, suggesting that the IOP-lowering effect of pregnancy is not enough alone. Approximately 10% of them show IOP elevation, uncontrolled IOP despite medical or surgical treatments, or glaucomatous damage progression [37,38].

A retrospective case series from the Harvard Medical School reviewed 28 eyes of 15 pregnant glaucomatous women and found that 57% of eyes had stable IOP and visual field (VF) damage, 18% showed an IOP increase without VF damage progression, and 18% of eyes experienced VF damage progression with stable or increased IOP and required additional medications to control the IOP [32]. In another study including a retrospective series of 13 eyes of eight pregnant women with pre-existing glaucoma, Mendez-Hernandez et al. reported that six patients required IOP-lowering medication to control the IOP and that only one patient showed IOP increase with VF loss progression [37]. A recent retrospective observational study, including 37 patients (67 eyes) of patients affected by POAG or normal-tension glaucoma who discontinued glaucoma medication during pregnancy, reported that 28.4% of eyes showed glaucoma morphological and/or functional damage progression after delivery (none during pregnancy) and that the glaucomatous damage progression was significantly associated with the mean IOP and IOP fluctuation values measured during pregnancy [38].

### 3.3. Labor and Delivery in OHT and Glaucomatous Women

Although clinical data are still lacking, labor and delivery may favor glaucoma damage progression, theoretically. Valsalva maneuvers during labor and vaginal delivery may induce IOP spikes and may precipitate acute angle-closure glaucoma in patients with narrow angles [39]; furthermore, a large amount of blood loss during vaginal delivery may potentially lead to transient systemic blood hypotension and ischemia, with increased risk of glaucomatous damage progression [1].

No specific guidelines exist regarding the delivery method, i.e., vaginal vs. Cesarian section, in glaucomatous pregnant women. Anyway, a recent survey of ophthalmologists and gynecologists regarding the choice of delivery mode in the presence of glaucoma reported that 16% of ophthalmologists indicated that they would recommend C-sections in pregnant women with “advanced or uncontrolled glaucoma”. In contrast, 25% of obstetrician–gynecologists would recommend a C-section for a pregnant woman with a “history of glaucoma” [40]. The study concluded by suggesting early delivery with C-section for patients with advanced or uncontrolled glaucoma who are expected to need multiple IOP-lowering medications or a glaucoma surgery [40].

Theoretically, a C-section may be preferred in advanced glaucomatous damage or trabeculectomy-operated patients because of the likelihood of damage progression or rupture of the bleb in operated patients [9].

## 4. The Glaucoma Medical Treatment During Pregnancy and Breastfeeding

Since elevated IOP is considered the most important and the only modifiable risk factor for the development and progression of glaucoma [1,2], IOP reduction represents the fundamental therapeutic strategy of current glaucoma management. This can be achieved with medical, laser, or surgical interventions [1]. However, hypotensive eye drops are the most used first-line treatment approach [1].

### 4.1. Drug Safety During Pregnancy and Breastfeeding: General Considerations

Locally or systemically, medications administered to pregnant or breastfeeding patients may have side effects on the pregnancy course, fetus, or newborn [10]. The global risk of serious congenital anomalies, preterm birth, and low birth weight has been estimated to account for approximately 3–8.6% of all neonates, and drugs and chemical exposure during pregnancy may increase this risk by only 1–3% [41].

Drug toxicity during pregnancy and nursing is related to the ability of the medication to cross the placenta and reach the fetus or to be excreted in the breast milk and be ingested by the newborn [10]. The possible side effects of medication are influenced by several variables, such as the administration route, molecular characteristics, dosage and exposure duration, pregnancy stage, fetal and newborn organ maturity, genetic characteristics of the mother and child, etc. [10]. IOP-lowering medications and other drugs used to treat OHT and glaucoma are commonly administered topically as eye drops. However, it has been demonstrated that 35–80% of the drug instilled in the conjunctiva can enter the systemic bloodstream (because of nasolacrimal duct drainage and conjunctival absorption), thus bypassing the hepatic metabolism [42]. If not degraded into the liver, some active drugs will be secreted into the breast milk and ingested by the newborn [10,43].

Drugs with a high ability to cross the placenta or be excreted in human breast milk are lipid-soluble, non-ionized, and not bound to plasma proteins, with low molecular weight (<700 Daltons to cross the placenta and <200 Daltons to be excreted in the breast milk) [10]. The maximum amount of drug found in human milk is 1–2% of the total administered maternal dose, although basic compounds may become slightly more concentrated in breast milk, which is more acidic (pH 7.2) than plasma (pH 7.4) [10,43]. Although potentially dangerous at any stage of pregnancy and nursing (Table 2), the drugs’ side effects may be greater during two delicate periods of pregnancy [10]:

- The first trimester of gestation, when most women may not even realize they are pregnant. It represents the period of organogenesis, i.e., the phase of maximal susceptibility to teratogens, especially between weeks 3 and 8, with higher risks of miscarriage, teratogenesis, and malformations;
- The last months of pregnancy, because the drugs may cross the placenta, reach fetal circulation, and interfere with the fetal or newborn’s cardiac, respiratory, and neurologic systems. Drug fetal exposure during the third trimester, particularly during the last 45 days, seems to be directly related to neonatal complications after birth.

**Table 2:** Potential risks of drug exposure at different pregnancy and nursing stages.

Stage	Potential Drug-Related Risks
Pre-conception and conception period	Fertility impairment
First trimester or phase of the organ’s differentiation or organogenesis	Miscarriage, embryo/fetus malformations, especially between weeks 3 and 8
Second trimester or phase of the increase in organ size	Reduced fetal growth, lower organ size, altered fetal metabolism
Third trimester or phase of the final fetal maturation and delivery preparation	Fetal malformations or premature labor
Labor and delivery	Premature or delayed labor, delivery complications
Post-natal and breastfeeding period	Newborn’s cardiac, neurological, respiratory, and metabolic abnormalities

Information derived from research on drug-associated risks to fetuses and neonates [10,43].

Moreover, some fetal characteristics may increase the drug’s toxicity, including small blood volume, an immature hepatic and kidney metabolism, and the drug’s excretion into the amniotic fluid from the kidneys, lungs, and skin [10]. Moreover, newborns may be particularly vulnerable to the undesirable effects of medications because of reduced drug metabolism and an immature blood–brain barrier [10]. Since randomized controlled trials are not feasible in pregnant and breastfeeding women because of clinical and ethical constraints, knowledge of drug safety in this population is generally based on animal studies, with results extrapolated for clinical applications in humans, spontaneous reporting of drug adverse events, or cohort studies in which a sufficient number of pregnant or nursing women are collected and compared to a control group [10,11,12].

In 1979, the United States (US) Food and Drug Administration (FDA) classified the risk of the drugs prescribed during pregnancy into five categories (A, B, C, D, X), where category A includes drugs whose safety has been demonstrated by RCTs and category X represents drugs with clearly demonstrated teratogenicity [44] (Table 3). The FDA pregnancy letter risk categories were updated in 2014 by the new “Pregnancy and Lactation Labeling Rule”, which includes information on pregnancy, breastfeeding, and exposure records [45]. To date, no drug used in ophthalmology conforms to category A or the new labeling standards. In particular, all IOP-lowering medications are labeled as category B, C, or D [11,12] and should be avoided unless the potential benefit to the patient justifies the potential risk to the fetus [5,8,9].

### 4.2. Safety of IOP-Lowering Medications During Pregnancy and Breastfeeding

Most IOP-lowering drugs have a molecular weight of 90–390 Daltons, can cross the placenta, and may be excreted in breast milk [11,12]. Table 4 summarizes the mechanisms of action and possible side effects of IOP-lowering medications during pregnancy and nursing.

Beta-adrenergic antagonists (beta-blockers) (Metipranolol, Timolol, Levobunolol, Betaxolol, Carteolol): Beta-blockers induce a diurnal IOP reduction of 20–25% by decreasing the aqueous humor production by the ciliary body epithelial cells [46]. They do not have any IOP-lowering effect during the nocturnal/sleep time due to the nocturnal decreased levels of endogenous circulating catecholamines [46]. Beta-blockers’ adverse effects include allergic conjunctivitis, keratitis, systemic systolic and diastolic hypotension, bradycardia, heart rate blood and oxygen saturation reduction, and bronchospasms [46]. Betaxolol, a selective beta-1-adrenoceptor antagonist, and carteolol, a non-selective beta-antagonist with intrinsic sympathetic activity, have less systemic respiratory and cardiovascular side effects in comparison with non-selective beta-blockers [46]. Beta-blockers are contraindicated in patients with asthma, chronic obstructive pulmonary disease, congestive heart failure, bradycardia, and heart block [46].

Beta-blockers are labeled as FDA pregnancy category C [44].

Systemic beta-blockers are the drugs most frequently used by obstetricians to manage hypertension during pregnancy or pre-eclampsia and have been associated with intrauterine growth inhibition, premature labor, and several side effects on newborns, including polycythemia, hypoglycemia, hyperbilirubinemia, transitory central nervous system (CNS) depression with lethargy, confusion and apnea, bronchoconstriction, and bradycardia and arrhythmia that may be transient or persistent, due to heart conduction defects [47]. Because of the association with cardiac, respiratory, and neurologic problems in newborns, it is recommended to discontinue the maternal therapy with systemic beta-blockers 2–3 days before delivery and to closely observe the newborns in the first 24–48 h after birth for bradycardia or other symptoms of beta-blockade [5,9].

Animal studies in mice, rats, and rabbits showed that a very high oral dose of timolol (approximately 7000 times the maximum recommended human ophthalmic dose) was not associated with fetal malformations [48].

The use of topical beta-blockers during pregnancy is still controversial. A population-based study including 244 pregnant women treated with topical glaucoma medications (189 with beta-blockers) compared with 1952 control pregnant women matched for age, year of delivery, maternal hypertension, and gestational diabetes reported that the risk of low birth weight in infants of mothers treated with topical beta-blockers was similar to that of the control group and significantly lower as compared with mothers treated with glaucoma medications other than beta-blockers [49].

Moreover, a large prospective observational study compared 75 pregnant glaucomatous women treated with topical timolol as monotherapy or in combination with other IOP-lowering drugs with 187 healthy pregnant women and did not find any influence of topical beta-blockers on pregnancy or fetal/neonatal malformations [50].

On the other hand, topical beta-blockers during pregnancy have been associated with some side effects, including fetal bradycardia and arrhythmia, delayed intrauterine growth, low birth weight, hypoglycemia, feeding difficulties, and apnea in newborns [37,51,52].

Considering the use of topical beta-blockers during nursing, previous studies have demonstrated that topical timolol and betaxolol are excreted in the human breast milk, where they reach a concentration 6 and 3 times higher than that in the plasma, with a maximum 90 min after topical instillation [53]. Their levels in human milk after eyedrop instillation is only 1/80 of the cardiac-effective dose and will not induce side effects unless the newborn’s hepatic or renal functions are impaired [53].

The American Academy of Pediatrics has approved using systemic and topical beta-blockers during nursing [54]; however, close monitoring of the infant’s cardiac and respiratory systems is recommended, especially for those with cardiopulmonary disease.

Prostaglandin (PG) F2α analogs and prostamide analogs (Latanoprost, Travoprost, Bimatoprost, and Tafluprost): These medications induce a diurnal and nocturnal IOP reduction of approximately 25–33% by increasing the uveo-scleral outflow and, to a lower extent, by increasing the trabecular meshwork outflow [55]. They have shown a high systemic safety profile, with no or rare associated systemic side effects during RCTs [55]. Local adverse effects include increased eyelash growth, periocular hyperpigmentation, palpebral and conjunctival hyperemia, allergic conjunctivitis, keratitis, and herpes virus activation. Because of their pro-inflammatory effect, their use is contraindicated in patients with active ocular fluorosis and cystoid macular edema [55].

All PG and prostamide analogs belong to the FDA pregnancy category C [44].

Latanoprost, travoprost, and bimatoprost (no data on tafluprost are available) are used systemically to induce premature labor or abortion because they can cross the blood-placenta barrier and cause the contraction of the uterine smooth muscles and the degradation of the corpus luteum [56]. Animal studies have demonstrated that very high doses of systemic PGs may have embryocidal effects, i.e., they may stimulate an abortion and teratogenic effects [57,58,59]. Experimental studies on pregnant rabbits have shown that latanoprost and bimatoprost may facilitate the delivery of nonviable fetuses at a dosage corresponding to 80 times and 40 times the ocular formulation, respectively [57]. The intramuscular administration of high doses of PG F2α in pregnant mice was associated with a high frequency of rib malformations [58]. Moreover, the treatment of pregnant rats with intravenous travoprost at doses over 250 times the maximum recommended ophthalmic human dose had a teratogenic effect, causing skeletal and visceral malformations [59].

The use of topical PG or prostamide analogs during pregnancy is still debated [60]. Previous authors have demonstrated that the plasma concentration after topical administration of latanoprost does not reach a sufficient level to stimulate non-ocular receptors and, in particular, to increase the uterine tone and induce abortion, premature labor, or fetal malformations in humans [61]. Moreover, a recent case series study using a large-scale longitudinal health database in the US (which includes more than 150 million enrollees between 2005 and 2020) quantified the percentage of spontaneous abortions amongst patients aged between 15 and 45 years that took a topical PG medication (3881 patients), compared with a random sample of women in the same age group and with similar risk profiles (3881 patients) [62]. The study found no association between the use of PG analogs during the first trimester of pregnancy and the risk of spontaneous abortion, which was 10% and 7% in the group assuming the PGs and in the control group, respectively [62].

A systematic review reported that the oral or vaginal use of misoprostol, a PG E1 analog, during the first trimester of pregnancy is linked with an increased risk of Moebius syndrome and limb defects [63]. Moreover, a multicenter pharmacovigilance study based on spontaneous report databases (the Japanese Adverse Drug Event Report database and the Food and Drug Administration Adverse Event Reporting System), conducted in 2022 to clarify the possible association between pregnancy loss and use of PG and prostamide analog eye drops in pregnant patients, found a total of 33 reports involving latanoprost, 23 involving bimatoprost, 13 involving travoprost, and 3 involving tafluprost, suggesting a likely association between spontaneous abortion and the use of latanoprost eyedrops in pregnant women [64].

PG and prostamide analogs have been demonstrated in animal breast milk [65], but it is unknown if they are present in human breast milk. Considering their rapid metabolism, with a half-life of approximately 17 min [61], it appears unlikely that they could have unwanted systemic side effects in newborns, especially when eye drops are instilled immediately after breastfeeding.

Latanoprostene bunod: It is a nitric oxide-donating prostaglandin F2α analogue recently approved by the US FDA, and it will be available in Europe soon [66]. It is metabolized into latanoprost, which increases the uveo-scleral outflow, and nitric oxide, which increases the trabecular outflow [66]. Latanoprostene bunod is effective in lowering the IOP both during the diurnal/wake and nocturnal/sleep periods, showing a lowering effect significantly higher than that of latanoprost and timolol, with a safety profile similar to that of PGs [66].

Latanoprostene bunod is labeled as FDA pregnancy category D [44]. When given systemically at high doses (over 20 times the human clinical dose), it has caused miscarriages, abortion, and fetal malformations in rabbits but not in rats [67]. Moreover, fetal toxicity has been noted in rabbits using an intravitreal dose very near the therapeutic margin [67].

Its efficacy and safety in human pregnancy and lactation have not yet been established.

Carbonic anhydrase inhibitors (CAIs): They inhibit the carbonic anhydrase isoenzyme 2, reducing aqueous ciliary body secretion, with a diurnal and nocturnal IOP decrease of approximately 15–20% for topical and 20–30% for oral administration [68,69]. Reported side effects include allergic dermatitis, corneal edema, Stevens–Johnson syndrome, anorexia, malaise, depression, and renal calculi [68]. Oral CAIs should be avoided in patients with chronic kidney disease [68].

CAIs are labeled as FDA pregnancy category C [44].

- Systemic CAIs (Acetazolamide and Methazolamide): Previous investigative studies have demonstrated the teratogenic effects of systemic acetazolamide in animals [70]. Systemic CAIs are frequently used in pregnant women for the treatment of idiopathic intracranial hypertension. Anyway, the Collaborative Perinatal Project [71], which included 1024 participants and other reports on pregnant women treated with oral acetazolamide to manage idiopathic intracranial hypertension [72], found no maternal complications or congenital disabilities. On the other hand, several case reports have associated the use of oral acetazolamide, especially during the first trimester of pregnancy, with side effects in the fetus and newborn, including fetal electrolytic disorders and renal dysfunctions, congenital malformations (ectrodactyly, syndactyly, oligodontia, and forelimb abnormalities), sacrococcygeal teratoma, low birth weight, neonatal electrolyte imbalance, and metabolic acidosis [73,74,75,76].
- Topical CAIs (Dorzolamide and Brinzolamide): High doses of systemic brinzolamide or dorzolamide, approximately 200–300 times the recommended topical human dose and 20 times the usual systemic therapeutic human dose, have a teratogenic effect, causing lower fetal weight and forelimb, kidney, and vertebral body malformations in rats, mice, hamsters and rabbits [77,78]. Although topical CAIs are generally considered safe in pregnancy [11,12], few reports have associated their use with the following side effects: intrauterine growth retardation, low birth weight, newborn renal tubular dysfunction, metabolic acidosis, and Peter’s anomaly [37,49,79]. Low (one-third of the maternal plasma levels) and likely non-harmful concentrations of CAIs have been found in the breast milk and plasma of newborns of mothers treated with oral acetazolamide [80]. They may potentially induce respiratory distress and renal or hepatic dysfunction, on the other hand. It is still unknown if topical CAIs are excreted in human breast milk, although both were found in rat milk [77,78].

Local and systemic CAIs treatments during lactation have been approved by the American Academy of Pediatrics [54].

Selective alpha-2-adrenoreceptor-agonist (Brimonidine): This drug has shown the ability to reduce IOP production and to improve the uveo-scleral outflow [46]. Its IOP-lowering effect, of approximately 20–25% [46], is effective during the diurnal/wake period, whereas it appears minimal during the nocturnal/sleep period [46]. Possible side effects are allergic and follicular conjunctivitis, dry mouth and nose, and systemic hypotension [46].

Brimonidine is the only antiglaucoma medication classified as FDA pregnancy category B [44]. Oral brimonidine given to pregnant rats and rabbits at very high doses, approximately 120–190 times the human therapeutic dose, caused maternal toxicity but no fertility impairment or fetal malformations [81].

Although topical brimonidine is considered safe during pregnancy [5,9], a study of 20 pregnant glaucoma patients treated with topical brimonidine delivered low-birth-weight newborns in 2 cases [49]. Moreover, a recent multi-center descriptive survey including 114 pregnancies of 56 patients affected by OHT or glaucoma treated with various topical IOP-lowering medications found a statistically significant association between the use of alpha-agonists during the third trimester of pregnancy and neonatal intensive care unit stay [82]. Topical brimonidine is excreted in the human breast milk [81] and, if present in the systemic bloodstream of children younger than 2 months of age, it can cross the blood–brain barrier, causing central nervous system depression, lethargy, seizures, apnea, hypotension, and bradycardia [83,84]. For this reason, it is recommended to discontinue its use during the third trimester or at least 30 days before delivery, and it is contraindicated in breastfeeding women and children affected by congenital or infantile glaucoma [5,9,11,12].

Parasympathomimetics or cholinergic agents or miotics (Pilocarpine and Carbachol): These drugs lower the IOP by traction of the scleral spur induced by ciliary muscle contraction, resulting in enhanced trabecular outflow [85]. The mean IOP reduction is approximately 20–25% [85]. Associated side effects are increased myopia, decreased visual acuity, cataract, periocular contact dermatitis, and ocular congestion [85].

Pilocarpine and carbachol are classified as pregnancy FDA category C [44]. In animal studies, they have been demonstrated to have teratogenic fetal effects [86]. One single study evaluating the use of systemic cholinergic drugs during the first 4 months of human pregnancy did not find any association with congenital abnormalities [87]. Pilocarpine may be excreted into the breast milk [43], but it is quickly metabolized when it reaches the bloodstream [85], so systemic side effects are unlikely. In newborns, miotics could theoretically induce gastrointestinal overactivity, salivation, sweating, nausea, tremors, and hypotension [85]. Moreover, previous case reports have associated the use of topical cholinergic agents with hyperthermia, seizures, and restlessness in neonates [88], so they should be avoided in breastfeeding patients [5,9,11,12].

Rho-associate protein kinase (ROCK) inhibitors (Netarsudil): Netarsudil is a potent Rho kinase/norepinephrine transporter inhibitor that has been recently released on the market [66]. It reduces IOP by approximately 10–20% by increasing the aqueous humor trabecular outflow because it causes the disruption of the cytoskeleton and the relaxation of the smooth muscle-like cells of the trabecular meshwork and Schlemm’s canal and by decreasing the episcleral venous pressure [66]. Its local side effects are eyelid erythema, conjunctival hyperemia (50–60% of cases), irritation, pruritus and discharge, subconjunctival hemorrhages, cornea verticillata, increased lacrimation, instillation-site pain, and blurred vision; anyway, no systemic adverse effects were observed [66]. The fixed combination of netarsudil–latanoprost, which has been recently approved for the treatment of OAG and OHT patients [66], provides an IOP-lowering effect of ≥30% and local side effects similar to its components [66].

ROCK inhibitors are pregnancy FDA category C [44]. Animal studies have demonstrated that the intravenous administration of high doses of netarsudil (approximately 125 times the plasma exposure at the recommended human ophthalmic dose) induces abortion in rats and mice but not in rabbits [89]. In contrast, it did not show clear teratogenic effects at physiologic concentrations in animal studies [90]. No data on using local netarsudil or fixed-association netarsudil–latanoprost in pregnant or breastfeeding women is available.

Hyperosmotic agents (mannitol, urea, isosorbide, and glycerol): Hyperosmotic agents are administered orally (isosorbide and glycerol) or intravenously (urea and mannitol) and induce hyperosmolarity in the bloodstream. In the presence of an intact blood–ocular barrier, this creates an osmotic gradient between intraocular and intravascular compartments, which results in a net passage of fluids from the aqueous and the vitreous body into the vascular space, with a fast IOP drop [91]. A secondary IOP-lowering mechanism seems to be the downregulation of the humor aqueous secretions mediated by the hypothalamic center that constantly controls fluids’ osmolarity with osmoreceptors [91]. The hyperosmotic agents are used almost exclusively in emergencies to manage acute IOP elevation, especially in cases of primary or secondary angle-closure glaucoma attacks. The systemic side effects of this medication class include fluid and electrolyte imbalance, metabolic acidosis, urinary retention, cerebral edema, headache, blurred vision, convulsions, nausea, vomiting, dehydration, heart failure, hypotension, and tachycardia [91]. They should be avoided in patients with severe dehydration, electrolyte abnormalities, heart or kidney failure, diabetic ketoacidosis, and hypersensitivity to the drug [91]. Hyperosmotic agents are included in FDA pregnancy category C [44]. No animal or human studies about the use of these drugs during pregnancy are available. Anyway, the intra-amniotic administration of mannitol and urea has a tocolytic effect [92], so their use during pregnancy should be considered with caution.

**Table 4:** The safety profile of IOP-lowering medications during pregnancy and breastfeeding.

Drug	Route	Systemic Side Effects	Local Side Effects	Contraindications	Toxicity in Pregnancy (Animal Studies)	Toxicity in Pregnancy (Humans)	US FDA Pregnancy Category	Recommendation During Pregnancy	Toxicity in Lactation (Humans	Recommendation During Nursing
Beta-adrenergic antagonists (beta-blockers) (Metipranolol, Timolol, Levobunolol, Betaxolol, Carteolol) [37,46,47,48,49,51,52]	Topical	Bradycardia, systolic and diastolic hypotension, heart rate reduction, blood oxygen saturation reduction, bronchospasm	Conjunctivitis, keratitis, corneal hypoesthesia	Patients with asthma, chronic obstructive pulmonary disease, congestive heart failure, bradycardia and heart block, hypersensitivity to the drug	None	Delayed fetal growth, fetal bradycardia and arrhythmia, low birth weight, newborn hypoglycemia, feeding difficulties, apnea	Category C or uncertain safe drugs	Possible use with control of fetal cardiac functions	Bradyarrhythmia, bronchospasm and central nervous system depression in newborns with impaired hepatic or renal functions	The use is approved by the American Academy of Pediatrics; avoid in newborns with cardiac or respiratory defects or with impaired hepatic or renal functions
Prostaglandin (PGs)F2α analogs and prostamide analogs (Latanoprost, Travoprost, Bimatoprost and Tafluprost) [55,56,57,58,59,63,64]	Topical	None or rare systemic hypertension	Increased eyelash growth, periocular hyperpigmentation, palpebral and conjunctival hyperemia, allergic conjunctivitis, keratitis, herpes virus activation	Patients with active ocular inflammation, cystoid macular edema, hypersensitivity to the drug	Abortion, skeletal and visceral malformations	Abortion, neonatal anomalies due to fetal brain ischemia, low birth weight	Category C or uncertain safe drugs	Use contraindicated during the first trimester; use with caution during last months; be sure that pregnant patient may recognize premature labor symptoms	Unknown	Use considered relatively safe because of the short drug half-time, especially with eyedrop instillation immediately after nursing
Latanoprostene bunod [66,67]	Topical	None	Increased eyelash growth, periocular hyperpigmentation, palpebral and conjunctival hyperemia, allergic conjunctivitis, keratitis, herpes virus activation	Patients with active ocular inflammation, cystoid macular edema, hypersensitivity to the drug	Abortion, fetal malformations	Unknown	Category C or uncertain safe drug	Avoid	Unknown	Avoid
Carbonic anhydrase inhibitors (Acetazolamide, Methazolamide) [70,73,74,75,76]	Systemic	Stevens–Johnson syndrome, anorexia, malaise, depression, renal calculi, allergic dermatitis,	None	Patients with chronic kidney disease, hypersensitivity to the drug	Lower fetal weight and forelimb, kidney and vertebral body malformations	Fetal electrolytic disorders and renal dysfunctions, congenital malformations (ectrodactyly, syndactyly, oligodontia and forelimb abnormalities), sacrococcygeal teratoma, low birth weight, neonatal electrolyte imbalance and metabolic acidosis	Category C or uncertain safe drug	Avoid during the first trimester	Respiratory distress and renal or hepatic dysfunction	The use is approved by the American Academy of Pediatrics
Carbonic anhydrase inhibitors (Dorzolamide, Brinzolamide) [37,49,77,78,79]	Topical	None	Allergic blepharitis and conjunctivitis, corneal edema	Hypersensitivity to the drug	Lower fetal weight and forelimb, kidney and vertebral body malformations	Intrauterine growth retardation, low birth weight, newborn renal tubular dysfunction, metabolic acidosis and Peter’s anomaly	Category C or uncertain safe drug	Avoid during the first trimester	Unknown	The use is approved by the American Academy of Pediatrics
Selective alpha-2-adrenoreceptor-agonist (Brimonidine) [46,49,81,82,83,84]	Topical	Dry mouth and nose, systemic hypotension	Allergic and follicular conjunctivitis	Hypersensitivity to the drug	None	Low birth weight	Category B or presumed safe drug	Avoid during the third trimester	Central nervous system depression, lethargy, seizures, apnea, hypotension, bradycardia	Avoid
Parasympathomimetics or cholinergic agents or miotics (Pilocarpine and Carbachol) [85,86]	Topical	Bradycardia, systemic hypotension, nausea, vomiting	Increased myopia, decreased visual acuity, cataract, periocular dermatitis, ocular congestion	Hypersensitivity to the drug	Fetal malformations	Unknown	Category C or uncertain safe drug	Avoid	Gastrointestinal overactivity, salivation, sweating, nausea, tremors, hypotension hyperthermia, seizures and restlessness in neonates	Avoid
Rho-associate protein kinase (ROCK) inhibitors (Netarsudil) [66,89]	Topical	None	Eyelid erythema, conjunctival hyperemia, irritation, pruritus and discharge, subconjunctival hemorrhages, cornea verticillata, increased lacrimation, instillation-site pain, blurred vision	Hypersensitivity to the drug	Abortion	Unknown	Category C or uncertain safe drugs	Avoid	Unknown	Avoid
Osmotic agents (mannitol, urea, isosorbide and glycerol) [91,92]	Systemic	Electrolyte imbalance, metabolic acidosis, urinary retention, cerebral edema, headache, blurred vision, convulsions, nausea, vomiting, dehydration, heart failure, hypotension, tachycardia	Flebitis, tissue necrosis	Severe dehydration or hypotension, electrolyte abnormalities, heart or kidney failure, diabetic ketoacidosishypersensitivity to the drug	Unknown	Unknown	Category D unsafe drug but possible benefits	Avoid	Unknown	Avoid

US FDA = United States Food and Drug Administration.

### 4.3. Safety of Other Drugs Used to Manage Glaucoma During Pregnancy and Breastfeeding (Brief Overview)

Eyedrop preservatives, including Benzalkonium chloride (BAK), which represents the most commonly used preservative in eyedrop formulations, are classified as FDA category C drugs, and no evidence of teratogenesis in humans is currently available [93,94].

All systemic and local anesthetics are included into FDA pregnancy category C [95,96,97,98], with the exception of local Lidocaine, Prilocaine, and Etidocaine, which were not associated with side effects in the mother or fetus in animal studies and are included in FDA pregnancy category B [97,98]; moreover, Lidocaine and Bupivacaine are considered safe during nursing [10]. Topical anesthetics, including Tetracaine, Proparacaine, and Oxybuprocaine eyedrops, were not associated with side effects on the mother or fetus. However, they should be used cautiously because no adequate animal or human studies data are available [10].

Antimetabolites, such as Mitomycin C and 5-fluorouracil, are FDA pregnancy category X teratogens because they have shown teratogenicity in animal models [99,100] and are therefore absolutely contraindicated in pregnancy [5,9]. However, no reports about their teratogenic effect in humans are yet available.

All topical antibiotics, including Chloramphenicol, Polymyxin B, Aminoglycosides, Fluoroquinolones, and Tetracyclines, are FDA pregnancy category C drugs [101,102,103], except for Erythromycin, which is an FDA pregnancy category B drug because of its poor ability to cross the human placenta, and it is regarded as a first-line therapy during pregnancy [101]. Moreover, both the American Academy of Pediatrics and the World Health Organization have approved the use of Erythromycin during nursing and as a first-line choice in the prophylaxis of the ophthalmia neonatorum caused by Chlamydia Trachimatis [54].

Systemic corticosteroids are labeled as FDA pregnancy category C [104], whereas topical corticosteroids, including Betamethasone, Fluorometholone, Prednisolone, Methylprednisolone, Dexamethasone, and Hydrocortisone, are classified as FDA category A because previous studies did not find any correlations between the use of ophthalmic CSs and teratogenicity in humans [105]. Prednisolone and Hydrocortisone have a lower ability to cross the placenta (in a percentage of approximately 10% of the plasma levels) than betamethasone, methylprednisolone, and dexamethasone (30–65% of the plasma levels) and may therefore have less side effects on the fetus [105]. Furthermore, both local and systemic corticosteroids are present in breastfeed milk and no side effects to the mother or newborn have been reported [104,105].

Topical non-steroidal anti-inflammatory drugs (NSAIDs) (Diclofenac, Flurbiprofen, Bromfenac, Indometacin, Ketorolac) [106], parasympatholytic agents (Atropine, Cyclopentolate, and Tropicamide) [10], and sympathomimetic agents (Phenylephrine) [10] are labeled as FDA pregnancy category D and should be avoided during pregnancy and lactation.

The safety of drugs used to manage glaucoma other than IOP-lowering medications during pregnancy and breastfeeding are summarized in Table 5.

## 5. The Glaucoma Laser Treatment During Pregnancy and Breastfeeding

Laser options per glaucoma treatment (Table 6) include the neodymium–yttrium–aluminum–garnet (Nd:YAG) laser iridotomy; diode laser cyclophotocoagulation; and laser trabeculoplasty, divided into argon laser trabeculoplasty (ALT), selective laser trabeculoplasty (SLT), and micropulse diode laser trabeculoplasty [107,108,109,110,111].

Reports describing a YAG laser iridotomy performed in a pregnant patient to treat an angle closure glaucoma have been published [108].

Diode laser cyclophotocoagulation has been described in a pregnant woman with refractory glaucoma [109].

Several successful ALT or SLT performed in planning-to-be-pregnant, pregnant or nursing women affected by POAG or OHT have been reported, resulting in a statistically significant reduction in IOP and/or the number of glaucoma eyedrops used post laser [112,113]. For example, a study including 40 eyes of 22 pregnant or breastfeeding women treated with SLT showed successful IOP lowering with a decreased number of anti-glaucoma medications used post laser [112]. Other authors treated 64 eyes of 32 pregnant women and 14 eyes of 7 planning-to-be-pregnant women affected by POAG with bilateral SLT and obtained good IOP control without medications during pregnancy and up to 6 months after delivery [113].

Laser trabeculoplasty has important limitations in childbearing women [107,110], including reduced efficacy in patients younger than 40 years; the need of an open and morphologically normal anterior chamber angle, whereas, unfortunately, congenital, secondary, or refractory glaucomas represent the most frequent types of glaucoma in young child-bearing patients [3]; the delayed onset of action, so that it cannot represent a good choice if urgent IOP lowering is required; the need for topical anesthetics and pre- and post-operative medications, which may be harmful for mother and fetus [10]; the short-term efficacy, which is overpassed by the short frame of the pregnancy itself anyway.

## 6. Glaucoma Surgical Treatment During Pregnancy and Breastfeeding

Surgery for glaucoma in pregnant and nursing women has a role when an uncontrolled IOP, despite maximal medical and laser treatments, is associated with VF damage progression or when the risk of increasing VF defects, in particular near the fixation center, appears to be very high [114]. Surgical options include the following:

- Filtration surgery: Trabeculectomy is the most frequently performed surgical procedure for glaucoma. It involves the creation of an alternative aqueous outflow pathway by constructing a fistula connecting the anterior chamber with the subscleral space and leading to the formation of a bleb of aqueous accumulation into the subconjunctival space [115]. In order to limit post-operative fibrosis and maintain the fistula’s patency, anti-metabolites such as Mitomycin C or 5-Fluorouracil are routinely applied on the sclera or under the conjunctiva intra- and/or post-operatively [114]. Trabeculectomy without antimetabolites has been described in pregnant patients [108,114,116,117]. Anyway, a very high failure rate of filtration surgery in pregnancy has been reported [114,116].
- Minimally invasive glaucoma surgery (MIGS): MIGSs are a group of varied surgical techniques and devices proposed to reduce the IOP with a high safety profile [118]. They include procedures that are “ab interno”, performed via a clear cornel incision, and “ab externo”, requiring a scleral or conjunctival incision, and based on their target, they may by classified into trabecular meshwork bypass, supraciliary shunts, and subconjunctival filtration devices [118]. As compared with the traditional filtration surgery, although showing a lower IOP-lowering effect and a shorter efficacy duration, MIGSs are characterized by a shorter surgical time, conjunctival sparing, and a reduced risk of lower intra-and post-operative complications and the number of post-operative medications [118]. All these characteristics could make MIGS particularly suitable for pregnant and nursing patients. The Xen gel 45 stent implant (Allergan, Dublin, Ireland) [119] and Ex-Press mini-shunt implant (Alcon, Fort Worth, TX, USA) [120] have been performed with good results in pregnant patients.
- Tube shunt surgery: It is a surgical procedure that consists of the implant of a tube shunt connecting the anterior chamber with the subscleral or subconjunctival space in order to allow the outflow of the aqueous humor [115]. Tube shunt implantation may be a good alternative when surgery is strictly necessary, especially considering the high rate of filtration surgery failure during pregnancy [114,116]. Aqueous drainage devices, including Ahmed and Baerveldt valves, have been successfully implanted in pregnant women [114,121,122].

Glaucoma surgery in childbearing women has several specific challenges that may explain its very high failure rate in this population [114,116]. Particular challenges of glaucoma surgery during pregnancy and nursing include the young patient’s age, which is related to faster would healing and incision and bleb fibrosis, with subsequent surgery failure [115]; the high proportion of congenital, juvenile, syndromic, or secondary glaucoma types that typically affect childbearing patients and that are associated with low glaucoma surgery success [3,115]; the need to avoid anti-metabolites, which are teratogens [99,100]; the high levels of vascular endothelial growth factors and placental growth factors typically found during pregnancy, which may favor would healing and fibrosis at the incision and bleb sites [123]; the difficulties in managing edematous tissues, especially during the second and third trimesters; the risk of deep maternal systemic hypotension and fetal ischemia due to the compression of the uterus on the aorta and the vena cava during a prolonged supine position, especially in the later stages of pregnancy [95]; the high risk of thromboembolism, requiring compression stockings or pneumatic compression; the risk of gastroesophageal reflux and aspiration pneumonia, especially during the third trimester; the possible side effects of anesthetics and of pre- and post-operative medications [10].

## 7. Discussion

The treatment of glaucoma in planning-to-be-pregnant, pregnant, and breastfeeding women represents a clinical challenge because it involves a delicate balance between the need to preserve the visual function of the mother and to avoid therapeutic strategies that may be potentially harmful to the fetus and newborn [4,5,6].

Although an IOP decrease is physiologically present in pregnant women [31] and several pieces of data suggest that estrogens may have an IOP-independent protective role against glaucoma onset and progression (Table 1) [35,36], the course of glaucoma during pregnancy may be highly variable, and approximately 10% of patients are at risk of IOP increase, uncontrolled IOP despite treatments, or glaucomatous damage progression [31,32]. Furthermore, glaucoma diagnosed in childbearing women is likely to be classified as congenital, juvenile, syndromic, or secondary, thus being frequently refractory to conventional treatments [3,9]. Glaucomatous pregnant women need, therefore, to be monitored at least once each trimester in mild to moderate cases and monthly in advanced cases [5,9]. Moreover, considering that the IOP values may be underestimated because of the corneal edema and the reduced corneal rigidity found during pregnancy [20,24], the close monitoring of optic nerve morphological and functional parameters seems to be mandatory [5,9].

In the absence of large-scale population studies, meta-analyses, and RCTs in humans providing precise established guidelines, the treatment of glaucoma during pregnancy or breastfeeding remains uncertain. A large survey of United Kingdom ophthalmologists conducted in 2007 reported that, amongst the 26% of participants declaring to have experience in treating glaucoma in pregnant women, 54% had continued the patient’s current medical therapy, 34% chose observation without any medication, and 12% had performed laser or surgery [13].

Medical therapy is the current treatment of choice in pregnant and lactating women, and topical beta-blockers seem to be the most frequently prescribed IOP-lowering medications during pregnancy and nursing, followed by prostaglandin and prostamide analogs [13,30,32,49,50]. For example, a survey including 605 British ophthalmologists showed that 45% of the participants considered beta-blocker eyedrops as a first-line therapy in pregnancy, whereas 33% prescribed prostaglandin analogs [13]. A large Asian population study including 244 pregnant women found that 77.5% were using a topical beta-blocker [49].

Glaucoma treatment in pregnant and breastfeeding women should be extremely individualized, based on several variables: the type and severity of the glaucoma; the rate of damage progression; the IOP level at which the damage has occurred; fellow eye status; the presence of a family history of severe glaucoma; the stage of pregnancy; the efficacy and safety profile of the different medical, laser and surgical options as supported by the existing literature. Moreover, the decision-making process should actively involve a well-informed patient together with a multidisciplinary team, composed of the ophthalmologist, family physician, obstetrics, neonatologists/pediatrics, and other healthcare providers.

As a general rule, anti-glaucoma therapy is indicated only for pregnant or nursing patients with moderate/advanced glaucoma who are at risk for developing functional impairment or decreasing their vision-related quality of life, or when the risks of progressive disease may outweigh the potential side effects of the treatment itself [5,8,9].

When possible, glaucoma management in childbearing women should begin before conception. Pre-conception is considered the ideal period in order to optimize medical therapy, to discuss the risks and benefits of different IOP-lowering medications, or to propose other therapeutic options, such as laser or MIGS, aiming to achieve good IOP control without or with the minimal number of drugs [5,7,9]. In general, all drugs should be avoided, if possible, at least during the first trimester of pregnancy, which represents the delicate period of the organogenesis, when the potential medication side effects could be more severe [10] (Table 2). Unfortunately, many pregnancies are unplanned or undiagnosed, so that the exposure to medications occurs often before pregnancy is recognized. For this reason, it appears extremely important to inform glaucomatous patients at childbearing age about the possible risks for pregnancy and fetuses associated with the different IOP-lowering medications or to propose alternative therapeutic approaches, such as SLT or MIGS, in order to reduce or stop medical therapy.

Limited clinical data are currently available about the safety of medical therapy during pregnancy and breastfeeding (Table 4 and Table 5). A recent multicenter survey including 114 pregnancies of 56 patients affected by glaucoma and treated with various topical IOP-lowering medications found an overall favorable safety profile, except for a statistically significant association between the use of alpha-agonists during the third trimester of pregnancy and neonatal intensive care unit stay [82].

A large retrospective cohort study, using data from a large healthcare database in Japan (between 2005 and 2018) and including 91 pregnant women who had received any IOP-lowering medications, did not find an increased risk of neonatal adverse outcomes, with similar results when beta-blockers and PG analogs were considered separately [30].

Previous studies have suggested that topical beta-blockers may have a higher safety profile in pregnancy. A recent population-based study including 244 pregnant women treated with topical glaucoma medications and compared with 1952 non-glaucomatous pregnant women showed a similar risk of low birth weight when beta-blockers were used, whereas the risk appeared significantly higher in infants born to mothers treated with glaucoma medications other than beta-blockers [49]. Moreover, a recent meta-analysis of previous studies found that the percentage of low birth weight could be 23% using CAIs, 7.1% for PGs and 8% for brimonidine, whereas the expected rate in the general population is of 6.2%, comparable to that observed using beta-blockers [50].

When medications are necessary, every effort should be made to minimize the drug’s systemic absorption. General rules for correct eyedrop instillation include the following: nasolacrimal occlusion or eyelid closure for 3–5 min immediately after instillation, which have been demonstrated to reduce systemic drug absorption over 60% [124]; the use of preservative-free eyedrops and fixed drug combinations when more than one drug is necessary; drug discontinuation before delivery; and eyedrop instillation just after nursing, because the drug levels in breast milk have been demonstrated to be highest at 30 to 120 min after eyedrop administration [53].

None of the currently available IOP-lowering medications are included in FDA pregnancy category A, i.e., demonstrated to be safe during pregnancy and nursing. Brimonidine is the only IOP-lowering medication actually labeled as FDA pregnancy category B, i.e., presumably safe (Table 4), and it should be considered as a first-line therapy during pregnancy, although it should be discontinued at least 30 days before delivery because of the risk of central nervous system and cardiac depression in newborns [82,83,84].

All the other IOP-lowering drugs are included into category C or D (Table 4); thus, their use should be avoided during pregnancy unless the potential benefit to the patient justifies the potential risk to the fetus [5,8,9].

Beta-blockers should be regarded as a second-line therapy. Although they are widely used in pregnancy to treat systemic hypertension [47], they are the most frequently used IOP-lowering medications in pregnancy [13,30,32], and have shown a good safety profile in population-based studies [49,50], they can alter fetal growth and heart rate or cause arrhythmia, bradycardia, hypotension, and CNS depression in newborns [37,51,52].

CAIs and PGs and prostamide analogs may represent a third-line therapy and should be limited to cases of uncontrolled IOP and clearly progressive glaucoma damage, when the IOP target is not reached with brimonidine or beta-blockers [3,6,8].

Although oral CAIs were not associated with maternal complications or fetus anomalies in large population-based studies [72,73], they have been linked to forelimb malformations and low birth weight [76], as well as to metabolic acidosis in the neonate [74,75] in some reports. Topical CAIs are teratogens (limb deformities) in animals [77,78] and have been associated with low birth weight and metabolic acidosis in newborns based on some case series [37,49,79]. Considering the incidence of limb deformities and low birth weight in both animal studies [70,77,78] and human case reports [73,74,75,76], it will be better to avoid both oral and topical CAIs during the first trimester, when the limbs are not already formed, and also during the last part of pregnancy, because CAIs may induce low birth weight, electrolytic imbalance, metabolic acidosis, respiratory problems, or the impairment of renal and hepatic function in newborns [37,49,73,79].

The use of topical PGs and prostamide analogs during pregnancy is still debated [60]. These drugs are FDA pregnancy category C and have demonstrated clear embryocidal and teratogenic effects in animals [57,58,59]. Although providing a good safety profile in previous studies [61,62], they have the ability to increase the uterine tone, reduce the perfusion to the fetus, and induce miscarriage or premature labor [56], and they have been associated with a higher risk of spontaneous abortion in a large multicenter pharmacovigilance study [64]. The most recent report by the American Glaucoma Society and Canadian Glaucoma Society recommended limiting the use of latanoprost, travoprost, and bimatoprost to the third trimester of pregnancy when any inducement of labor will be safe [5].

Cholinergic agents have demonstrated teratogenic effects in animals [86] and are poorly tolerated in young patients because of their several side effects [85], so that they are better avoided in childbearing women [5,9].

Latanoprostene bunod, which is the only IOP-lowering medication labeled as FDA pregnancy category D [67], and ROCK inhibitors, labeled as FDA pregnancy category C [89,90], should be avoided during pregnancy because their effects on pregnancy are already unknown [5,9].

Beta-blockers and CAIs are considered the first-line therapy during nursing [54] and also the first-line choices in infants with congenital glaucoma [9]. Anyway, the administration of both drugs requires a careful clinical and laboratory assessment of the newborn. Beta-blockers have been associated with bradyarrhythmia, bronchospasms, and CNS depression in newborns [37,51,52], and CAIs may induce electrolytic imbalance, metabolic acidosis, respiratory problems or the impairment of renal and hepatic function in newborns [37,49,73,79].

Because of their rapid metabolism [61], PGs are considered a reasonable option during breastfeeding, especially when applied immediately after nursing. Anyway, no evidence is present to support the use of PGs during nursing [6]. Brimonidine should be absolutely avoided in the last months of pregnancy and during breastfeeding [6,8,11] because it can cause CNS depression, lethargy, seizures, apnea, hypotension, and bradycardia [83,84]; moreover, its use during the third trimester of pregnancy has been associated with neonatal intensive care unit stay [82].

No evidence of the teratogenesis of eyedrop preservatives is actually available. Anyway, because of the possible unknown side effects [10], the preservative-free forms of the IOP-lowering medications seem to be the better choice in pregnant and breastfeeding patients.

With regard to drugs used pre-, intra-, and post-operatively for the laser or surgical treatment of glaucoma (Table 5), general anesthesia may cause maternal hypoxia, hypercapnia, systemic hypotension, and fetal cardiovascular and CNS depression [95], and it should be avoided during pregnancy and nursing. Topical anesthesia combined with subconjunctival or sub-Tenonian anesthesia induce less systemic drug absorption than retrobulbar anesthesia and may be preferred in pregnant women [98].

Local corticosteroids are labeled as FDA pregnancy category A and are considered safe during both pregnancy and breastfeeding [104,105]. Prednisolone should be preferred because of its lower ability to cross the placenta [105].

Amongst topical antibiotics, erythromycin is an FDA pregnancy category B drug and it is considered as a first-line therapy during both pregnancy [101] and nursing [54].

Topical non-steroidal anti-inflammatory drugs are contraindicated during pregnancy because of their teratogenic effects [106], whereas the American Academy of Pediatrics has approved their use during breastfeeding because of their low concentrations found in human breast milk [54]. The use of mydriatics is contraindicated during the first trimester of pregnancy, although tropicamide is preferred for its short duration of action both as a diagnostic or therapeutic option [10].

Considering the role of laser treatments for glaucoma during pregnancy and breastfeeding, laser trabeculoplasty, especially SLT, may theoretically provide the least amount of risk to the mother and fetus/newborn when compared with other glaucoma therapeutic approaches. It may therefore represent a reasonable alternative to treat glaucoma in pregnant and nursing patients to avoid or minimize topical and/or systemic IOP-lowering drugs and their potential harmful side effects and could be particularly useful in the pre-conceptional period [5,6,7,9]. Moreover, laser procedures have several advantages over incisional surgery because they require only topical anesthesia and limited pre- and post-operative medications, are performed in an upright position, and are outpatient procedures with fast rehabilitation [107,110,111]. Unfortunately, laser treatments have low success rates in young patients and require an open and morphologically normal irido-sclero-corneal angle to be performed [107,110,111], so that they may have limited indications in young childbearing patients, where congenital, secondary, or refractory glaucomas represent the most frequent glaucoma types [3].

Laser cyclophotocoagulation, filtration, and tube shunt surgery are generally reserved to patients with moderate–severe glaucomatous damage and may be proposed when an uncontrolled IOP, despite maximal medical and laser treatments, is associated with demonstrated or highly likely visual field damage progression, especially near the fixation center [107,114,115,116]. Anyway, glaucoma surgery in childbearing women shows very poor success rates [114,116] and several challenges [114,116], including the possible side effects of anesthetics and of pre- and post-operative medications [10] and the risk of deep systemic hypotension due to a prolonged supine position, especially in the later stages of pregnancy, which may compromise fetal circulation [95].

MIGSs, especially “ab interno” procedures, are generally considered less invasive than filtration surgery and may be a reasonable alternative therapeutic approach to glaucoma in childbearing patients. MIGSs have the advantages of smaller incisions, shorter operating times, and minimal intra- and post-operative complications and medications, and they may be performed under local anesthesia. For all these characteristics, they may be extended to the pre-conceptional planning or proposed to pregnant or nursing patients in order to stop or minimize medical therapy [118]. Anyway, it is recommended to defer surgery after the first trimester to avoid potentially embryocidal or teratogenic effects of the anesthetics and pre- and post-operative medications [10,95,98]. Moreover, surgery should be avoided in the third trimester because of difficulties of anesthesia, positioning, tissue handling and healing, fetal distress, premature labor, and low birth weight [114,116]. For all these reasons, the second trimester seems to be the best period for surgical interventions.

### 7.1. Limitations of Current Knowledge

The most important limitation of the available knowledge about the safety of the therapeutic approaches to manage glaucoma in pregnant and nursing women is the lack of large prospective randomized controlled trials that are not feasible in this population for both clinical and ethical reasons [125]. Indeed, most of the currently available evidence that may help clinicians is based on animal studies, which are frequently unreliable in demonstrating teratogenicity in humans, or case reports, where it is often difficult to discriminate the side effects of the drugs and the complications related to pregnancy or underlying diseases [125]. Other confounding factors related to glaucomatous pregnant or breastfeeding patients may be their non-adherence to the therapy due to the fear of the drug-related teratogenic risk or, on the contrary, their underestimation of the possible adverse effects on the fetus/newborn of the topical administration of eyedrops [125].

In the absence of evidence-based data, the potential teratogenicity of most therapeutic agents in pregnant and nursing women remains largely unknown [10]. In particular, none of the actually available IOP-lowering medications can be considered safe in pregnancy and breastfeeding [11,12] (Table 4), so that their prescription in this population always represents an off-label use.

### 7.2. Future Therapeutic Approaches

Several drug delivery systems for sustained glaucoma therapy are actually under development, including punctal plugs, contact lenses, implants, and depot systems placed in the extraocular, periocular, and intraocular regions (intracameral, supraciliary, intravitreal) [126]. At the present moment, the Bimatoprost sustained-release implant, approved by the FDA in 2020, is the first biodegradable intracameral sustained released implant proposed for reducing the IOP in patients with POAG or OHT, which will be marketed as Durysta (Allergan plc, Dublin, Ireland) [126]. A previous study has demonstrated that the intracameral delivery system of Bimatoprost requires a very low total dose of the drug to achieve a superior sustained IOP decrease as compared with Bimatoprost eyedrops for at least 4 months [127].

Other promising intracameral biodegradable implants, still under evaluation for safety and efficacy, are Envisia Therapeutics’ ENV515 travoprost implant (Envisia Therapeutics, Research Triangle Park, NC, USA), Glaukos’ iDose^™^ (Glaukos Corporation, Aliso Viejo, CA, USA), Ocular Therapeutix’s OTX-TIC travoprost implant (Ocular Therapeutix Inc, Bedford, MA, USA), and Santen’s polycaprolactone implant with PGE2-derivative DE-117 (Santen, Osaka, Japan). Other prostaglandin-based sustained delivery systems include Allergan’s bimatoprost ring (placed in the conjunctival fornix) (Allergan, Group Abbvie, Pringy, France), Ocular Therapeutics’ OTX-TP intracanalicular travoprost implant a liposomal formulation of latanoprost for subconjunctival injection, and the PGE2 derivative PGN 9856-isopropyl ester that is applied to the periorbital skin (Ocular Therapeutix Inc, Bedford, MA, USA) [128].

Sustained delivery systems, especially the intracameral and intravitreal ones, have the advantage to increase medical adherence, to deliver the drug near the tissue involved in the glaucoma pathology (trabecular meshwork or retinal ganglion cells), and to reduce the systemic absorption of the medication and the drug exposure to off-target tissues [126,127,128], which may be extremely interesting in pregnant and breastfeeding women.

## 8. Recommendations for the Management of Glaucoma During Pregnancy and Breastfeeding

These recommendations are based on a synthesis of the existing literature and are divided according to the different pre-conceptional, gestational, and nursing periods.

Pre-conceptional period
  - Evaluate intraocular pressure (IOP) control and morphological and functional glaucomatous damage.
  - Inform the patient about the benefits and risks related to the different treatment modalities.
  - Personalize the treatment based on the patient’s risk profile given a pregnancy.
  - Explain the methods to minimize systemic drug absorption.
  - Propose selective laser trabeculoplasty (SLT) or minimally invasive glaucoma surgery (MIGS) in order to stop or minimize IOP-lowering drugs.
  - Obtain a stable IOP before pregnancy.

First trimester
  - Stop any medications when possible, with close monitoring of the IOP and visual field.
  - Propose SLT in order to stop or reduce medications.
  - Prescribe topical Brimonidine as a first-line option.
  - Prescribe topical beta-blockers as a second-line option.
  - Prescribe topical carbonic anhydrase inhibitors (CAIs) or Prostaglandin (PG) analogs as a third-line option as part of a maximal medical therapy if strictly necessary.
  - Avoid systemic CAIs if not strictly necessary.
  - Avoid miotics, Latanoprostene bunod, and Netarsudil.
  - Avoid surgery, excepted in cases of uncontrolled IOP with clearly progressive glaucomatous damage.

Second trimester
  - Stop any medications when possible, with close monitoring of the IOP and visual field.
  - Propose SLT in order to stop or reduce medications.
  - Prescribe topical Brimonidine as the first-line option.
  - Prescribe topical beta-blockers as a second-line option, closely monitoring the fetal heart rate and growth.
  - Prescribe topical CAIs as a third-line option as part of a maximal medical therapy if strictly necessary.
  - Prescribe topical PG analogs as a third-line option as part of a maximal medical therapy, if strictly necessary, after educating patients about the symptoms of premature labor.
  - Prescribe systemic CAIs only in severe or refractory cases, with strict control of the fetal growth.
  - Avoid miotics, Latanoprostene bunod, and Netarsudil.
  - Choose this trimester of pregnancy to perform surgical interventions if planned (prefer MIGS).

Third trimester
  - Stop any medications when possible, with close monitoring of the IOP and visual field.
  - Propose SLT in order to stop or reduce medications.
  - Prescribe topical CAIs as the first-line option.
  - Prescribe topical beta-blockers as a second-line option, closely monitoring the fetal heart rate and growth.
  - Consider systemic CAIs as a third-line option in severe or refractory cases, with strict control of fetal growth.
  - Avoid Brimonidine (newborn’s life-threatening central nervous system depression) and PG analogs (labor progression impairment).
  - Avoid miotics, Latanoprostene bunod, and Netarsudil.
  - Avoid surgery, except in cases of uncontrolled IOP with clearly progressive glaucomatous damage.

Breastfeeding
  - Stop any medications when possible, with close monitoring of the IOP and visual field.
  - Educate the patients to assume topical therapy immediately after nursing.
  - Propose SLT to stop or reduce medications.
  - Prescribe topical beta-blockers or CAIs as first-line therapy, avoiding beta-blockers in newborns affected by congenital cardiac or pulmonary problems.
  - Consider systemic CAIs as a second-line therapy in selected cases.
  - Prescribe PG analogs as a third-line therapy as a part of a maximal medical therapy if strictly necessary.
  - Avoid Brimonidine (newborn’s life-threatening central nervous system depression).

## 9. Conclusions

The management of glaucoma during pregnancy and breastfeeding presents several specific challenges. In the absence of clearer recommendations and more effective therapies, physicians must depend on personalized care plans that include vigilant monitoring, conservative treatment choices, and collaboration with a multidisciplinary team to safeguard the welfare of both the mother and child.

Pre-conception is the ideal period in order to optimize medical therapy or to propose other therapeutic options, such as laser or MIGS, aiming to achieve good IOP control before the beginning of the pregnancy.

All medications should be avoided, if possible, during the first trimester of pregnancy, i.e., during organogenesis, and minimized during the last months of pregnancy, because they may cross the placenta and affect the fetal and newborn’s cardiac, respiratory, and neurologic system function.

Although no topical or systemic IOP-lowering medications have clear evidence of safety during pregnancy and lactation, topical brimonidine or beta-blockers may be acceptable in pregnant women, but both drugs should be stopped at least 30 days before delivery and substituted by local CAIs. CAIs and beta-blockers are allowed during nursing.

Traditional surgery (filtration or shunts) may be considered in selected cases at high risk of damage progression, and it should be performed preferably during the second trimester, because of the possible adverse effects of anesthesia and post-op medications.

SLT and MIGS, especially when performed during the pre-conception period, may offer on option to reduce or avoid the use of anti-glaucoma medication in selected cases, whereas their short-term efficacy is overpassed by the short frame of the pregnancy itself.

Several drug delivery systems for sustained glaucoma therapy are actually under development, which may be extremely interesting in pregnant and breastfeeding women because they may reduce the systemic absorption of the medication and the drug exposure to off-target tissues.

With the rise in maternal age and the increasing diagnosis of glaucoma among women of reproductive age, the profession of ophthalmology must emphasize the development of safer pharmacological and surgical solutions specifically designed for this demographic. Further studies and clinical data need to be shared in order to enable healthcare providers to preserve maternal vision while protecting fetal and infant health when managing glaucoma during pregnancy and nursing.

## Figures and Tables

**Table 1:** Changes affecting intraocular pressure and optic nerve during pregnancy.

Variables	Mechanisms	Results
> estrogens	> arterial vasodilation → systemic hypotension > arterial vasodilation → > ocular and cerebral blood flow	< aqueous production →IOP reduction Optic nerve protection against ischemic damage
	> venous vasodilation → < venous pressure > collagen synthesis → < lamina cribrosa deformability RGCs protection against IOP-related damage	> aqueous trabecular and uveo-scleral outflow → IOP reduction Optic nerve protection against mechanical damage Optic nerve neuroprotection
	> systemic water retention → corneal oedema	IOP underestimation
> relaxin	> collagen degradation → > corneal deformability	IOP underestimation
	> collagen degradation → > trabecular permeability	> aqueous trabecular outflow → IOP reduction
	> collagen degradation → > scleral permeability	> aqueous uveo-scleral outflow → IOP reduction
> progesterone	< effect of endogenous corticosteroids	> aqueous trabecular outflow → IOP reduction
> HCG	> cyclic adenosine monophosphate in the ciliary body	< aqueous production → IOP reduction
> metabolic acidosis	> arterial vasodilation → systemic hypotension	< aqueous production → IOP reduction
	> venous vasodilation → < venous pressure	> aqueous trabecular and uveo-scleral outflow → IOP reduction

IOP = intraocular pressure; HCG = human chorionic gonadotropin. Data derived from pertinent literature regarding physiological changes of IOP and optic nerve during pregnancy [22,23].

**Table 3:** United States (US) Food and Drug Administration (FDA) classification of the risks related to the drugs prescribed during pregnancy [44].

Category A or safe drugs	Controlled studies in humans have shown no risk to the fetus.
Category B or presumed safe drugs	Animal studies have shown no risk to the fetus, but there are not adequate studies in pregnant women. Animal studies have shown risks to the fetus, but none of the adverse side effects seen in animal studies were confirmed in pregnant women.
Category C or uncertain safe drugs	Animal studies are inadequate or have shown adverse effects on the fetus, and there are no controlled studies on pregnant women.
Category D or unsafe drug but possible benefits	Investigational or post-marketing data have shown some risks to the fetus in pregnant humans, but potential benefits may outweigh the potential risks.
Category X or unsafe drug	Drugs are definitely contraindicated in pregnancy because studies in animals or humans or post-marketing reports have shown fetal risks that clearly outweigh any benefit to the patient.

**Table 5:** Safety profile of drugs other than IOP-lowering medications used to manage glaucoma during pregnancy and breastfeeding.

Drug Family	Drug	Route	Toxicity in Pregnancy (Animal Studies)	Toxicity in Pregnancy (Humans)	US FDA Pregnancy Category	Recommendation During Pregnancy	Toxicity in Lactation (Humans)	Recommendation During Nursing
Eyedrop preservatives [93,94]	Benzalkonium chloride (BAK)	Topical	abortion, low birth weight and size and minor sternum malformations	Unknown	Category C or uncertain safe drug	Avoid	Unknown	Avoid
General anesthetics [95,96]	Phenobarbital, Etomidate, Propofol	Systemic	Fetal malformations	Maternal hypoxia, hypercapnia, systemic hypotension, fetal ischemia due to maternal hypotension, fetal cardiovascular and central nervous system depression, premature delivery, low birth weight, neural tube defects	Category C or uncertain safe drug	Avoid	Unknown	Avoid
Local anesthetics [97,98]	Lidocaine, Prilocaine, Etidocaine	Local	None	Unknown	Category B or presumed safe drug	Possible use	None	Possible use
Local anesthetics [98]	Bupivacaine, Mepivacaine	Local	Fetal malformations	Fetal bradycardia and central nervous system depression	Category C or uncertain safe drug	Avoid	Unknown	Avoid
Topical anesthetics [12]	Tetracaine, Proparacaine, Oxybuprocaine	Topical	Unknown	Unknown	Category C or uncertain safe drug	Use with caution	Unknown	Use with caution
Antimetabolites [99,100]	Mitomycin C, 5-Fluorouracil	Topical, Local	Fetal malformations	Unknown	Category X or definitely unsafe drug	Avoid	Unknown	Avoid
Antibiotics [101]	Erythromycin	Topical	None	Fetal hepatotoxicity	Category B or presumed safe drug	Possible use (first-line antibiotic therapy)	None	Approved by the American Academy of Pediatrics and by the World Health Organization for use in nursing
Antibiotics [101,102]	Chloramphenicol	Topical	Unknown	Grey baby syndrome and bone marrow suppression	Category C or uncertain safe drug	Avoid during the last part of pregnancy	Vomiting and meteorism	Avoid
Antibiotics [101]	Polymyxin B	Topical	Unknown	Fetal neurotoxicity and nephrotoxicity	Category C or uncertain safe drug	Avoid	None	Possible use
Antibiotics [101]	Aminoglycosides (Netilmicin, Neomycin, Gentamicin, Tobramycin)	Topical	Unknown	Fetal ototoxicity and nephrotoxicity	Category D or unsafe drug but possible benefits	Avoid	Newborn ototoxicity and nephrotoxicity	Avoid
Antibiotics [101,103]	Fluoroquinolones	Topical	Unknown	Possible agenesis, mutagenesis and carcinogenesis	Category D or unsafe drug but possible benefits	Avoid	Unknown	Avoid
Antibiotics [101]	Tetracyclines	Topical	Unknown	Inhibition of bone growth	Category D or unsafe drug but possible benefits	Avoid	Inhibition of bone growth and color alterations in deciduous teeth (with changes to brown)	Avoid
Corticosteroids [104]	Betamethasone, Fluorometholone, Prednisolone, Methylprednisolone, Dexamethasone, Hydrocortisone	systemic	Fetal malformations	Unknown	Category B or presumed safe drug	Prefer prednisolone and hydrocortisone	None	Possible use
Corticosteroids [105]	Betamethasone, Fluorometholone, Prednisolone, Methylprednisolone, Dexamethasone, Hydrocortisone	Topical	None	None	Category A or safe drugs	Prefer prednisolone and hydrocortisone	None	Possible use
Non-steroidal anti-inflammatory drugs (NSAIDs) [106]	Diclofenac, Bromfenac, Indometacin, Ketorolac, Flurbiprofen	Topical	Fetal malformations	Fetal renal impairment, premature closure of the ductus arteriosus	Category D or unsafe drug but possible benefits	Avoid	None	Approved by the American Academy of Pediatrics for use in nursing (ibuprofen, naproxen, and indomethacin)
Parasympatholytics [10]	Atropine, Cyclopentolate, Tropicamide	Topical	Fetal malformations	Fetal bradycardia, minor fetal malformations	Category D or unsafe drug but possible benefits	Avoid, prefer Tropicamide when necessary	Unknown	Avoid, prefer Tropicamide when necessary
Sympathomimetics [10]	Phenylephrine	Topical	Fetal malformations	Minor fetal malformations, newborn renal failure due to general vasoconstriction	Category D or unsafe drug but possible benefits	Avoid	Unknown	Avoid

US FDA = United States Food and Drug Administration.

**Table 6:** Laser options for glaucoma management.

Neodymium–yttrium–aluminum–garnet (Nd:YAG) laser iridotomy: this laser procedure is indicated in cases of narrow-angle or angle-closure glaucoma in order to create a communication between the anterior and the posterior chambers, allowing for aqueous deflux [107].
Diode laser cyclophotocoagulation: this procedure causes the partial disruption of ciliary body processes, and it could be considered as an alternative to traditional incisional glaucoma surgery [107].
Argon laser trabeculoplasty (ALT): argon laser burns are focalized on the anterior trabecular meshwork, causing both structural and coagulative damage [107].
Selective laser trabeculoplasty (SLT): laser-induced thermal damage is confined to the pigmented area of the irradiate tissue [110,111]. SLT has largely substituted ALT in glaucoma management because it induces minimal thermal damage to the trabecular meshwork with similar efficacy [110,111].
Micropulse diode laser trabeculoplasty: it has been proposed to avoid the thermal spread of laser energy with consequent coagulative damage [107].
